# Endoscopic ultrasound‐guided fine‐needle biopsy for the diagnosis of gastric metastasis from breast cancer mimicking primary linitis plastica: A case report

**DOI:** 10.1002/deo2.115

**Published:** 2022-04-05

**Authors:** Kenta Yamada, Junichi Kaneko, Moeka Watahiki, Yuya Ida, Megumu Koda, Kyoichi Fukita, Yu Takeshita, Kenichi Takahashi, Masaki Takinami, Atsushi Tsuji, Masafumi Nishino, Yurimi Takahashi, Yuzo Sasada, Takanori Yamada

**Affiliations:** ^1^ Division of Gastroenterology Iwata City Hospital Shizuoka Japan; ^2^ Division of Hepatology Iwata City Hospital Shizuoka Japan

**Keywords:** breast cancer, endoscopic ultrasound‐guided fine‐needle biopsy, Franseen‐tip needle, gastric metastases, linitis plastica

## Abstract

For gastric lesions in a patient with a history of breast cancer, it is essential to distinguish between primary gastric cancer and gastric metastasis from breast cancer. However, gastric metastasis from breast cancer often mimics primary linitis plastica, and histological diagnosis may be difficult with conventional endoscopic biopsies. Herein, we describe the case of a 75‐year‐old woman who presented at our hospital with epigastralgia and vomiting. She had a history of mastectomy for carcinoma of the right breast and had received hormone therapy as adjuvant therapy. Computed tomography at arrival showed thickening of the gastric wall at the antrum and peritoneal dissemination. Esophagogastroduodenoscopy showed mucosal swelling of the antrum and stenosis of the pylorus, and histological diagnosis failed with conventional endoscopic biopsies. Endoscopic ultrasound‐guided fine‐needle biopsy using a Franseen needle was performed, and a diagnosis of gastric metastasis from breast cancer was made. She received hormone therapy and chemotherapy after deployment of a metallic stent for gastric outlet obstruction. To the best of our knowledge, this is the first case of gastric metastasis from breast cancer diagnosed using endoscopic ultrasound‐guided fine‐needle biopsy.

## INTRODUCTION

Breast cancer frequently metastasizes to the lymph nodes, lungs, bones, liver, and brain; however, metastasis to the gastrointestinal system, particularly to the stomach, is rare.^1^ Gastric metastasis from breast cancer can mimic primary gastric cancer, especially gastric linitis plastica (GLP), and require pathological confirmation for diagnosis.^2^, ^3^ However, lesions resembling GLP may not be histopathologically diagnosed with conventional endoscopic biopsy because tumor cells are more abundant in the submucosa than in the mucosa. We herein present the case of a patient with gastric metastasis that resembled GLP, who was diagnosed by endoscopic ultrasound‐guided fine‐needle biopsy (EUS‐FNB) using a Franseen needle.

## CASE REPORT

A 75‐year‐old woman was referred to our hospital with complaints of epigastralgia and vomiting. She also presented with anasarca and a weight loss of 10 kg in a month. She had a history of mastectomy for carcinoma of the right breast at the age of 72 years. Pathological analysis of the right breast cancer showed an infiltrating lobular carcinoma, and 12 of 22 axillary nodes were affected. Immunohistochemical analysis revealed the following results: estrogen receptor (ER) positivity, progesterone receptor negativity, and human epidermal growth factor receptor type 2 positivity. Following mastectomy, she received oral administration of letrozole 2.5 mg/day as adjuvant therapy.

**FIGURE 1:**
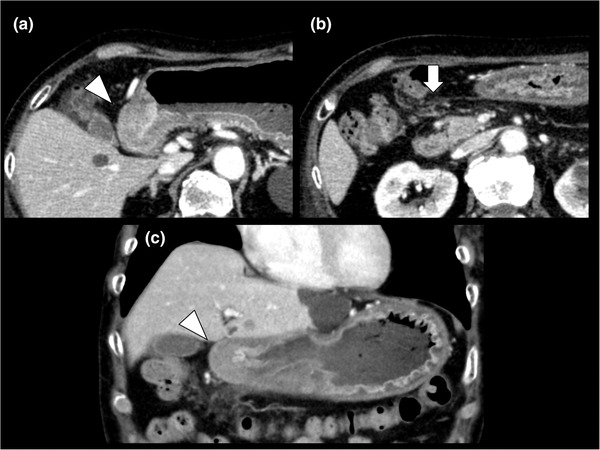
Computed tomography (CT) findings at arrival to the hospital: (a) Axial CT scan showing gastric wall thickening at the antrum (arrowhead); (b) axial CT scan showing scattered nodules in the peritoneum (arrow); (c) coronal CT scan showing gastric wall thickening at the antrum (arrowhead)

**FIGURE 2:**
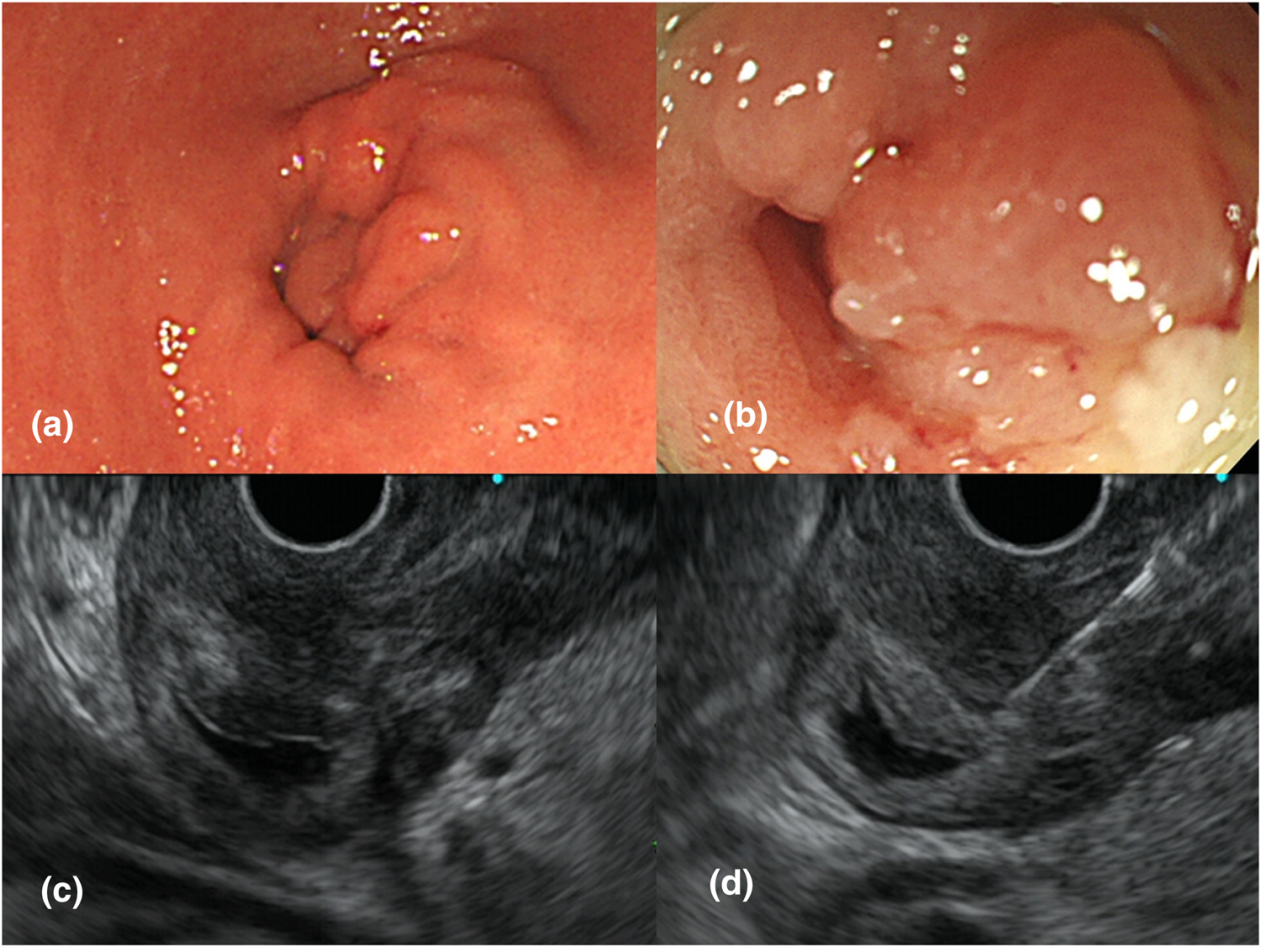
Endoscopic findings: (a, b) esophagogastroduodenoscopy showing mucosal swelling of the antrum and a stenotic site at the pylorus; (c) endoscopic ultrasonography findings showing gastric wall thickening at the antrum; (d) endoscopic ultrasound‐guided fine‐needle biopsy

**FIGURE 3:**
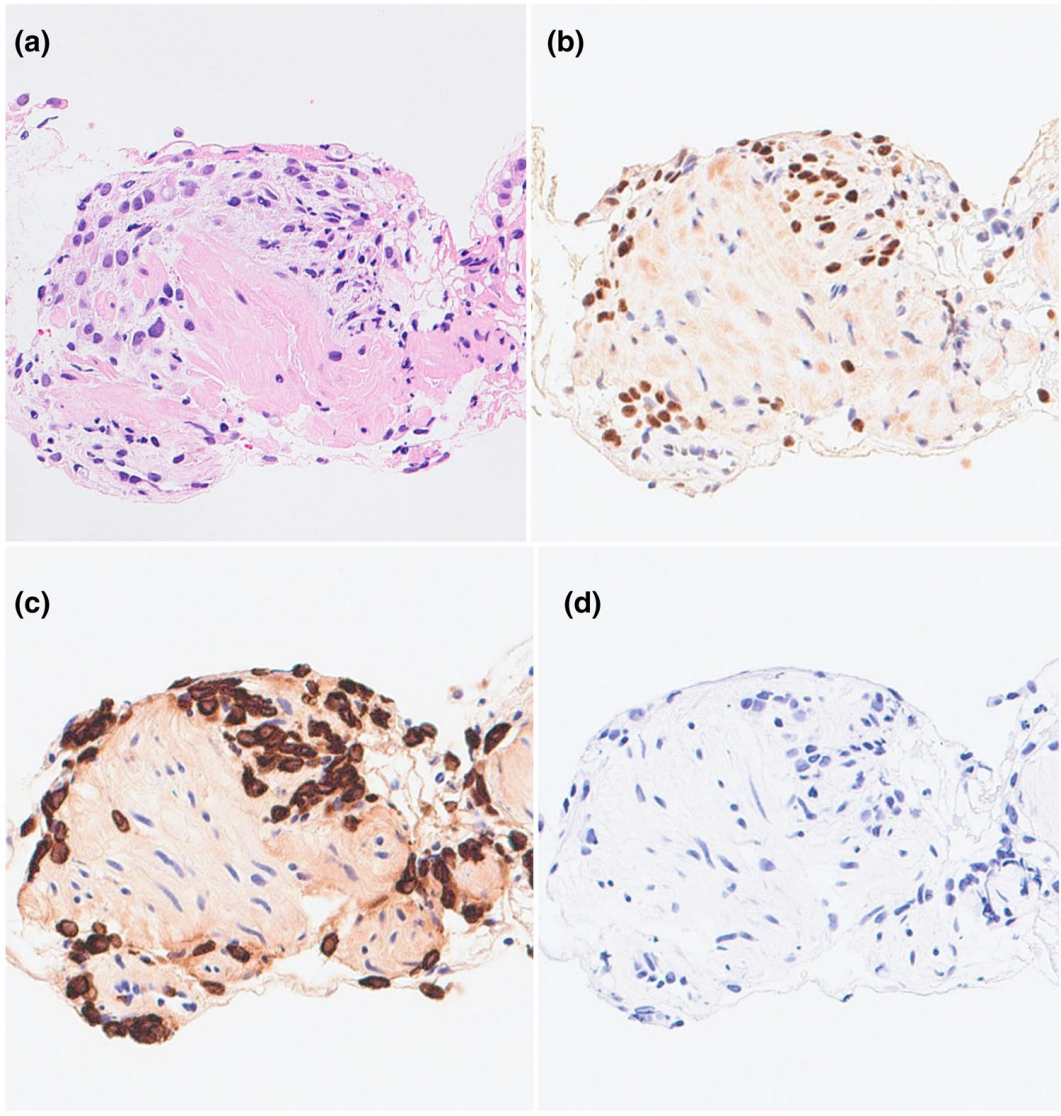
Histopathological findings of an endoscopic ultrasound‐guided fine‐needle biopsy specimen: (a) hematoxylin and eosin staining; (b) positive immunohistochemistry staining for estrogen receptor; (c) positive immunohistochemistry staining for cytokeratin 7; (d) negative immunohistochemistry staining for cytokeratin 20

Abdominal computed tomography performed at arrival to the hospital showed thickening of the gastric wall at the antrum and scattered nodules in the peritoneum around the stomach (Figure 1). Esophagogastroduodenoscopy (EGD) revealed food retention in the stomach, mucosal swelling of the antrum, and pyloric stenosis. In addition, the EGD equipment did not pass through the stenotic site (Figure 2). Thus, it was suspected that a malignant tumor was causing gastric outlet obstruction (GOO). However, the conventional endoscopic biopsy did not indicate any malignant cells. The serum carcinoembryonic antigen (CEA), carbohydrate antigen (CA) 125, and CA15‐3 levels were within the normal range. Additional EGD and several biopsies were performed; however, a pathological diagnosis could not be made. Therefore, we performed EUS‐FNB for the pathological diagnosis. EUS findings indicated that the gastric wall at the antrum thickened up to 7 mm. A 22‐gauge Franseen needle (Acquire; Boston Scientific Corporation, Natick, MA, USA) was used for EUS‐FNB. Gastric wall thickening was punctured under EUS guidance (Figure 2 and Supplementary Video). The needle was moved back and forth 10 times under 10 ml of negative pressure on each pass, and a total of four passes were performed. On macroscopic on‐site evaluation, white tissue, considered as visible core tissue, was sampled.^4^ Histological findings showed that the gastric stroma was infiltrated by the small carcinoma cells. Immunohistochemistry findings indicated a positive result for ER and cytokeratin (CK) 7 and a negative result for CK 20 in the biopsy specimen (Figure 3). Based on the pathological results, she was diagnosed with gastric metastases from breast cancer with peritoneal dissemination.

**FIGURE 4:**
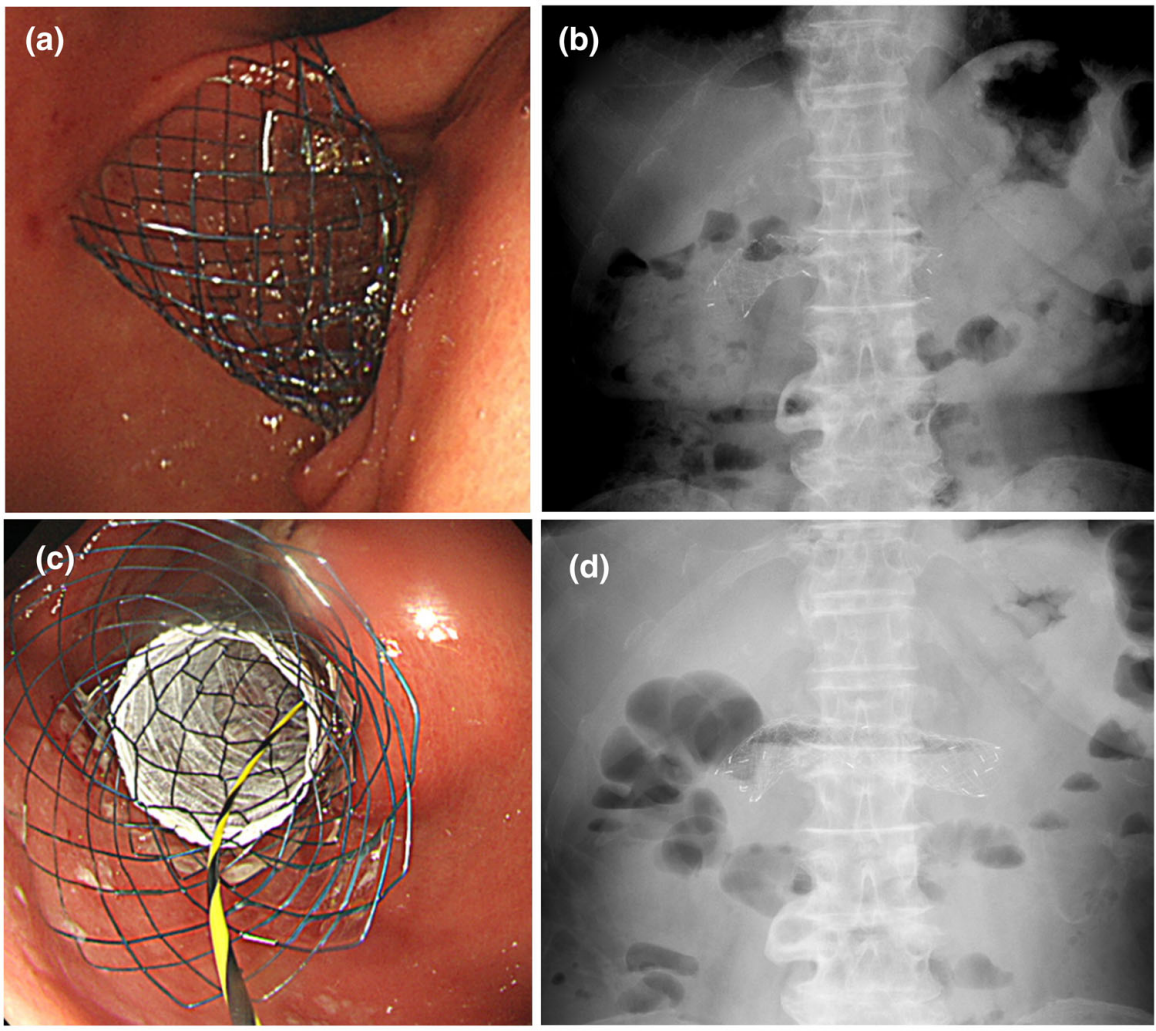
Endoscopic and radiographic images of the metallic stent; (a) the endoscopic images after metallic stent placement; (b) the radiographic image shows that the metallic stent was fully open; (c) the endoscopic images after additional metallic stent placement; (d) the radiographic image shows that the additional metallic stent was fully open

An uncovered self‐expandable metallic stent (SEMS) (Niti‐S Pyloric Duodenal D‐type Stent; Taewoong Medical, Seoul, Korea) measuring 22 mm in diameter and 12 cm in length was placed at the stenotic site (Figure 4ab). SEMS placement alleviated GOO and allowed oral intake. However, 1 month later, GOO symptoms recurred due to tumor ingrowth, and a covered SEMS (Niti‐S Pyloric Duodenal D‐type Stent; Taewoong Medical) measuring 20 mm in diameter and 12 cm in length was placed (Figure 4c,d). She subsequently had been on hormone therapy and chemotherapy for over 6 months without recurrence of GOO symptoms; she was alive at 7 months after diagnosis, with slight tumor progression.

## DISCUSSION

In our case, the patient, who had a history of breast cancer, developed GOO due to gastric wall thickening. Using EUS‐FNB and a Franseen needle, she was successfully diagnosed with gastric metastasis from breast cancer, although a prior endoscopic biopsy was negative for malignancy.

Gastric metastasis from breast cancer is rare, with a reported incidence of 0.3% according to a previous study^3^; however, this is probably an underestimation because endoscopy is performed primarily for symptomatic patients. Invasive lobular cancer is the most common histological type of primary breast cancer, followed by invasive ductal carcinoma. Most gastric metastases from breast cancer were often diagnosed more than 2 years after the diagnosis of primary breast cancer and presented with non‐specific symptoms, such as abdominal pain, anorexia, and vomiting.^1^ From endoscopic findings, the most common presentation was GLP, and pathological consequences are essential in distinguishing between gastric metastasis and primary gastric cancer.^3^ In GLP lesions, tumor cells are abundant in the submucosa, resulting in gastric wall thickening. GLP lesions have high false‐negative rates with conventional endoscopic biopsy; thus, sampling from GLP lesions remains challenging.^5^ A recent retrospective study showed that EUS‐guided tissue acquisition (EUS‐TA) using a standard FNA needle and Procore needle (Cook Medical Inc., Limerick, Ireland) had a sensitivity of 86% (19/22) in patients with gastric wall thickening and prior negative conventional biopsy.^6^ Recently, new biopsy needles, such as Franseen‐type and fork‐tip needles, have been designed to procure samples with preserved tissue architecture.^7^ These new needles can procure larger samples than standard needles, resulting in the availability of immunological staining and next‐generation sequencing. Takahashi et al. reported two patients with GLP, in which EUS‐FNB with a Franseen needle was used to successfully perform immunological staining and confirm diagnosis after a failed conventional endoscopic biopsy.^8^ Currently, 19‐gauge, 22‐gauge, and 25‐gauge Franseen needles are available. In general, finer needles are more flexible and easier for puncturing; whereas, they tend to obtain a smaller sample. A prospective, randomized, non‐inferiority trial on pancreatic and peripancreatic masses showed that a 25‐gauge Franseen needle was inferior to the 22‐gauge needle in procuring a histologic core.^9^ Therefore, a 22‐gauge or 19‐gauge needle may be preferable for diagnosing gastric metastasis from breast cancer. In our case, EUS‐FNB using a 22‐gauge Franseen needle aided in successfully making a diagnosis of gastric metastasis from breast cancer. To the best of our knowledge, this is the first report of a case in which gastric metastasis from breast cancer was diagnosed using EUS‐TA.

It is essential to distinguish between primary gastric cancer and gastric metastases from breast cancer in patients with a history of breast cancer. For patients with primary gastric cancer, surgical resection is the most effective treatment for patients without distant or peritoneal metastases. On the other hand, patients with gastric metastasis from breast cancer are indicated for systemic therapy with chemotherapy and hormone therapy, and surgery is not an option except in atypical situations, such as GOO or uncontrolled bleeding. Traditionally, surgical gastrojejunostomy (sGJJ) has been performed for GOOs. However, endoscopic gastroduodenal stent placement using a SEMS has recently been used as an alternative treatment to sGJJ. A previous prospective randomized trial showed that SEMS placement allows earlier resumption of food intake, shorter hospitalization period, and lower cost compared with sGJJ, Meanwhile, sGJJ showed better results with fewer recurrent obstructive symptoms and reinterventions.^10^ Thus, SEMS placement may be preferred, especially for patients with a poor prognosis and condition. In our case, endoscopic gastroduodenal stent placement was performed for GOO because of peritoneal dissemination and poor condition. GOO symptoms recurred 1 month later, but they were resolved with an additional SEMS placement, after which the patient successfully received hormone therapy and chemotherapy without recurrence of GOO symptoms during the observation period.

In conclusion, it is essential to distinguish between gastric metastasis from breast cancer and primary gastric cancer when endoscopists encounter gastric lesions in patients with a history of breast cancer. EUS‐FNB using a Franseen needle can be useful for the diagnosis of gastric metastasis mimicking primary gastric cancer, especially GLP.

## CONFLICT OF INTEREST

The authors declare no conflicts of interest.

## FUNDING INFORMATION

None

## ETHICS STATEMENT

Not applicable.

## Supporting information




**Supplementary Video**: Endoscopic ultrasonography‐guided fine‐needle biopsy of gastric wall thickening.
